# Prognostic Value of Peripheral Inflammatory Markers in Preoperative Mucosal Melanoma: A Multicenter Retrospective Study

**DOI:** 10.3389/fonc.2019.00995

**Published:** 2019-10-09

**Authors:** Yixi Wang, Hao Zhang, Yuhan Yang, Tao Zhang, Xuelei Ma

**Affiliations:** ^1^Department of Biotherapy, West China Hospital and State Key Laboratory of Biotherapy, Sichuan University, Chengdu, China; ^2^West China School of Medicine, Sichuan University, Chengdu, China; ^3^Department of Pancreatic Surgery, West China Hospital, Sichuan University, Chengdu, China; ^4^State Key Laboratory of Biotherapy and Cancer Center, West China Hospital, Sichuan University, Chengdu, China

**Keywords:** mucosal melanoma, prognosis, NLR, PLR, LMR

## Abstract

**Background:** Peripheral neutrophil-to-lymphocyte ratio (NLR), platelet-to-lymphocyte ratio (PLR), and lymphocyte-to-monocyte ratio (LMR) have been widely reported prognostic predictors for many cancers. However, data predicting prognosis on mucosal melanoma is currently limited. This study aimed to identify the value of these inflammatory markers in predicting prognosis in preoperative mucosal melanoma.

**Methods:** In this multicenter retrospective study, we assessed patients with preoperative mucosal melanoma for 7 years. Connection between baseline inflammatory markers (NLR, PLR, and LMR) and overall survival (OS) and progression-free survival (PFS) was analyzed by Kaplan–Meier curve with a log-rank test. Then, NLR, PLR, and LMR, along with characteristics of patients, were included in the univariate and multivariate Cox hazards regression model to examine the correlation with OS and PFS. The optimal cutoff value of these inflammatory markers was stratified by receiver operating characteristic (ROC) curve.

**Results:** Patients with baseline NLR > 3.07, PLR > 118.70, or LMR ≤ 7.38 had significantly poorer OS and PFS according to Kaplan–Meier curve with a log-rank test. Univariate analysis indicated that surgery, alkaline phosphatase (ALP), NLR, PLR, and LMR were statistically connected to both OS and PFS. In multivariate analysis, LMR (hazard ratio [HR] = 0.113; 95% CI: 0.017–0.772; *P* = 0.026) and surgery (HR = 0.166; 95% CI: 0.033–0.846; *P* = 0.031) maintained significant relevance with OS.

**Conclusions:** This research revealed that a higher NLR and PLR and a lower LMR than the cutoff point was associated with a worse prognosis of preoperative mucosal melanoma. Thus, we assumed that NLR, PLR, and especially LMR were potential prognostic predictors of preoperative mucosal melanoma.

## Introduction

Mucosal melanoma is a rare and aggressive malignant tumor that includes head and neck, gastrointestinal, gynecological, urological, and respiratory tract melanomas. It is distinct from melanomas originated from other sites of the body, making up <2% of all melanoma (1). In a great measure, mucosal melanomas are confirmed at a relatively advanced clinical stage and correlated with a poor outcome and 5-year overall survival (OS) rate of 25% (2).

In recent years, increasing evidence has indicated that systematic inflammation participates in the initiation, progression, and metastasis of tumors (3). The inflammatory response can be identified by several parameters in peripheral blood, for instance, baseline leukocytes and their subtypes, C-reactive protein (CRP), plasma fibrinogen, neutrophil-to-lymphocyte ratio (NLR), and lymphocyte-to-monocyte ratio (LMR), and all of those were discussed as prognostic indicators in plenty of solid tumors (4–7).

In melanoma, elevated neutrophil (8, 9) and monocyte (10) counts, with either in the presence of a higher NLR (11–13), were reported as predictors of poorer survival in melanoma at clinical advanced stage and in patients receiving immunotherapies. Those researches concerned the whole family of melanomas, although mucosal melanoma is epidemiologically and genetically distinct from other subtypes of melanomas, and they also differ in the responses to different forms of therapy (1, 2). Specifically, raised serum lactate dehydrogenase (LDH) was documented to be significantly predictive for mucosal melanoma (14). However, less is known about the effectiveness of peripheral inflammatory cell ratios as prognostic factors in mucosal melanoma, including NLR, LMR, and platelet-to-lymphocyte ratio (PLR). Moreover, peripheral inflammatory cell ratios are readily available, easy to examine, and economical. Hence, we performed analyses of patients with preoperative mucosal melanoma of any stage, with the purpose of identifying the prognostic value of peripheral inflammatory markers in mucosal melanoma.

## Methods

### Patients

All 40 preoperative mucosal melanoma patients were retrospectively recruited from three medical institutions between October 2010 and July 2017, including West China Hospital of Sichuan University, Chengdu China, Tibet Chengdu Branch of West China Hospital of Sichuan University, Chengdu, China, and The Forth People's Hospital of Chengdu, Chengdu China. Subjects were selected according to the following inclusion criteria: (1) histologically confirmed diagnosis of mucosal melanoma (head and neck, gastrointestinal, gynecological, urological, and respiratory tract melanomas) within 3 months before inclusion even if they hospitalized for other non-cancer diseases and (2) had at least one eligible and available preoperative blood test, restricted to peripheral blood test conducted without resection of primary or metastatic tumor, biopsy of lymph nodes, or any other medical treatment for mucosal melanoma. Patients were excluded based on the following: (1) they had non-mucosal melanomas or other cancer; (2) they received any treatment for mucosal melanoma; (3) they were unavailable for preoperative blood test; (4) they had infection or blood transfusion within 3 months before the diagnosis of mucosal melanoma; or (5) they had a history of chronic infection or autoimmune diseases. Our research was approved by the ethics committee of West China Hospital of Sichuan University. We claim that this study was conducted in accordance with the principles of the Declaration of Helsinki. However, because of the retrospective nature of the study, patient consent for inclusion was waived. Also, the data and information of participants we collected all came from routine examination and treatment of this disease.

### Data Collection

Characteristics of patients and preoperative blood test results were retrieved for each eligible patient from clinical records of the host institutions. Collected characteristics included age, sex, surgery, chemical therapy, and radiotherapy and metastasis. Preoperative blood test results included counts of neutrophils, lymphocytes, monocytes, platelets, together with levels of hemoglobin (Hb), albumin (Alb), alkaline phosphatase (ALP), and LDH. In addition, inflammatory markers were defined as follows: NLR = neutrophil/lymphocyte; PLR = platelet/lymphocyte; and LMR = lymphocyte/monocyte.

### Outcomes

OS of each eligible patient was the primary endpoint, determined as the interval from the first pathological diagnosis to either death caused by any reason (event) or the final follow-up (censored). Progression-free survival (PFS) was considered as the second outcome, identified as the duration between the first pathological diagnosis and disease progression or death from any cause when last follow-up was end.

### Procedures

All enrolled patients were assigned to two divisions according to the optimal cutoff point of NLR, PLR, and LMR, respectively, calculated by receiver operating characteristic (ROC) curve based on OS. The follow-up of each patient was obtained from clinical records, phone calls, and e-mails, terminated on April 17, 2018.

### Statistical Analysis

The main variables, NLR, PLR, and LMR, were stratified by the optimal cutoff point based on analytic results of ROC curve. All the clinical data retrieved and Hb, Alb, ALP, and LDH levels were defined as categorical variables and analyzed using the chi-squared test and the Fisher's exact test when necessary. Characteristics potentially associated with NLR, PLR, and LMR were analyzed by univariate analysis with Cox proportional hazards model. Then, a multivariate analysis was performed to test characteristics with *P* < 0.05 from the previous univariate analysis and other potential confounding factors. To analyze the correlation between inflammatory marker ratios (NLR, PLR, and LMR) and OS and PFS, a Kaplan–Meier curve with a log-rank test was conducted. All statistical analyses were performed using SPSS version 22.0 (IBM Corporation, Armonk, USA).

## Results

### Patients Characteristics

Initially, 96 patients were recruited, of whom 47 (49%) had a preoperative blood test. After excluding seven patients with recurrent mucosal melanoma, we included 40 (42%) eligible patients in the final analysis. Among all subjects, 35.0% (14/40) of patients were aged over 65 years, with a median age of 58 years, while 65.0% (26/40) of patients were female. The median follow-up time was 1434.50 days (range: 235–2,666 days). At the clinical endpoint, 42.5% (17/40) of patients were dead and 57.5% (23/40) of patients were alive. Meanwhile, no any loss of follow-up occurred. For treatment, 90.0% (36/40) of patients had surgery, 35.0% (14/40) received chemotherapy, and 22.5% (9/40) underwent radiotherapy. The tumor of 47.5% (19/40) of patients was metastatic.

### Inflammatory Markers and Clinical Characteristics

We applied the ROC curve to examine the sensitivity and specificity of respective NLR, PLR, and LMR thresholds for OS and PFS. Consequently, the proportion under the curve was 0.705 [95% confidence interval (CI): 0.515–0.894], 0.729 (95% CI: 0.565–0.892), and 0.215 (95% CI: 0.053–0.376), with cutoff points of 3.07, 118.70, and 7.38, respectively. All characteristics of patients grouped by levels of inflammatory markers are presented in Table 1. No statistical significance was found within groups stratified by NLR level in all features (*P* > 0.05). More patients receiving chemotherapy were observed as patients with PLR > 118.70 than patients with PLR ≤ 118.70 (*P* = 0.028). More male patients were observed as patients with LMR > 7.38 than patients with LMR ≤ 7.38 (*P* = 0.026). For groups stratified by PLR, no statistically significant difference was observed among gender, age, surgery, metastasis, radiotherapy, and Hb, PLT, Alb, ALP, and LDH levels (*P* > 0.05). Similarly, no statistical significance was found among age, surgery, metastasis, chemotherapy, radiotherapy, and Hb, PLT, Alb, ALP, and LDH levels stratified by LMR level (*P* > 0.05).

**Table 1:** Comparison among 40 patients with preoperative mucosal melanoma based on NLR, PLR, and LMR groups.

	**Total *n* (%)**	**NLR**			**PLR**			**LMR**		
		**≤3.07 (*n* = 29)**	**>3.07 (*n* = 11)**	***P*-value**	**≤118.70 (*n* = 18)**	**>118.70 (*n* = 22)**	***P-*value**	**≤7.38 (*n* = 21)**	**>7.38 (*n* = 19)**	***P*-value**
**Gender**, ***n*** **(%)**										
Male	14 (35.0)	7 (24.1)	7 (63.6)	0.049	4 (22.2)	10 (45.5)	0.125	4 (19.0)	10 (52.6)	0.026
Female	26 (65.0)	22 (75.9)	4 (36.4)		14 (77.8)	12 (54.5)		17 (81.0)	9 (47.4)	
**Age**, ***n*** **(%)**										
<65	26 (65.0)	18 (62.1)	8 (72.7)	0.795	10 (55.6)	16 (72.7)	0.257	14 (66.7)	12 (63.2)	0.816
≥65	14 (35.0)	11 (37.9)	3 (27.3)		8 (44.4)	6 (27.3)		7 (33.3)	7 (26.8)	
**Surgery**, ***n*** **(%)**										
No	4 (10.0)	2 (6.9)	2 (18.2)	0.637	0 (0.0)	4 (18.2)	0.168	1 (4.8)	3 (15.8)	0.527
Yes	36 (90.0)	27 (93.1)	9 (81.8)		18 (100.0)	18 (81.8)		20 (95.2)	16 (84.2)	
**Metastasis**, ***n*** **(%)**										
No	21 (52.5)	16 (55.2)	5 (45.5)	0.538	12 (66.7)	9 (40.9)	0.105	12 (57.1)	9 (47.4)	0.536
Yes	19 (47.5)	13 (44.8)	6 (54.5)		6 (33.3)	13 (59.1)		9 (42.9)	10 (52.6)	
**Chemotherapy**, ***n*** **(%)**										
No	26 (65.0)	20 (69.0)	6 (54.5)	0.629	15 (83.3)	11 (50.0)	0.028	15 (71.4)	11 (57.9)	0.370
Yes	14 (35.0)	9 (31.0)	5 (45.5)		3 (16.7)	11 (50.0)		6 (28.6)	8 (42.1)	
**Radiotherapy**, ***n*** **(%)**										
No	31 (77.5)	22 (75.9)	9 (81.8)	1.000	16 (88.9)	15 (67.2)	0.238	17 (81.0)	14 (73.7)	0.865
Yes	9 (22.5)	7 (24.1)	2 (18.2)		2 (11.1)	7 (31.8)		4 (19.0)	5 (26.3)	
Hb^*^, ***n*** **(%)**										
< LLN^†^	4 (10.0)	2 (6.9)	2 (18.2)	0.637	0 (0.0)	4 (18.2)	0.168	1 (4.8)	3 (15.8)	0.527
≥LLN^†^	36 (90.0)	27 (93.1)	9 (81.8)		18 (100.0)	18 (81.8)		20 (95.2)	16 (84.2)	
**PLT**^‡^, ***n*** **(%)**										
<300	36 (90.0)	27 (93.1)	9 (81.8)	0.637	18 (100.0)	18 (81.8)	0.168	21 (100.0)	15 (78.9)	0.091
≥300	4 (10.0)	2 (6.9)	2 (18.2)		0 (0.0)	4 (18.2)		0 (0.0)	4 (21.1)	
**Alb^§^**, ***n*** **(%)**										
<35	37 (92.5)	28 (96.6)	9 (81.8)	0.178	18 (100.0)	19 (86.4)	0.305	20 (95.2)	17 (89.5)	0.928
≥35	3 (7.5)	1 (3.4)	2 (18.2)		0 (0.0)	3 (13.6)		1 (4.8)	2 (10.5)	
**ALP^||^** **(UI/L)**, ***n*** **(%)**										
<150	38 (95.0)	29 (100.0)	9 (81.8)	0.071	18 (100.0)	20 (90.9)	0.492	21 (100.0)	17 (89.5)	0.219
≥150	2 (5.0)	0 (0.0)	2 (18.2)		0 (0.0)	2 (9.1)		0 (0.0)	2 (10.5)	
**LDH**^¶^ **(UI/L)**, ***n*** **(%)**										
<245	36 (90.0)	27 (93.1)	9 (81.8)	0.637	16 (88.9)	20 (90.9)	1.000	19 (90.5)	17 (89.5)	1.000
≥245	4 (10.0)	2 (6.9)	2 (18.2)		2 (11.1)	2 (9.1)		2 (9.5)	2 (10.5)	

* Hb, hemoglobin;

† LLN, lower limits of normal, 120 g/L for male adults and 110 g/L for female adults;

‡ PLT, platelet;

§ Alb, albumin;

|| ALP, alkaline phosphatase;

¶ *LDH, lactate dehydrogenase*.

### Inflammatory Markers and Prognosis

**Figure 1:**
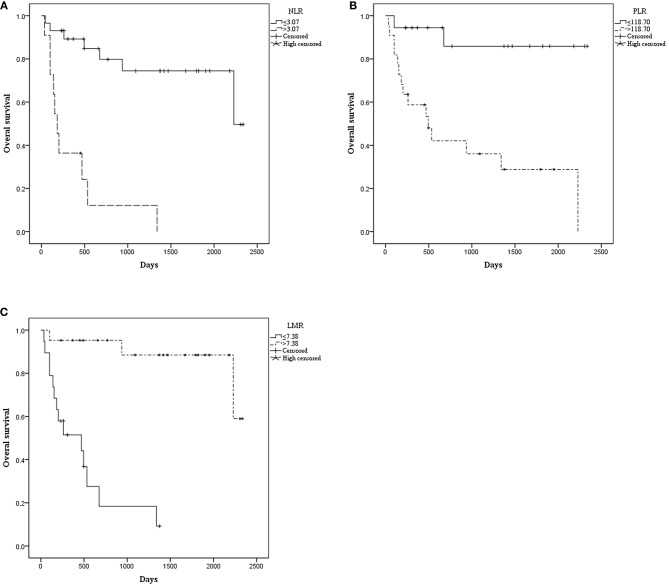
Kaplan–Meier curve for overall survival (OS) of 40 patients with pretreated mucosal melanoma stratified by inflammatory makers: **(A)** OS stratified by neutrophil-to-lymphocyte radio (NLR); **(B)** OS stratified by platelet-to-lymphocyte radio (PLR); **(C)** OS stratified by lymphocyte-to-monocyte radio (LMR).

**Figure 2:**
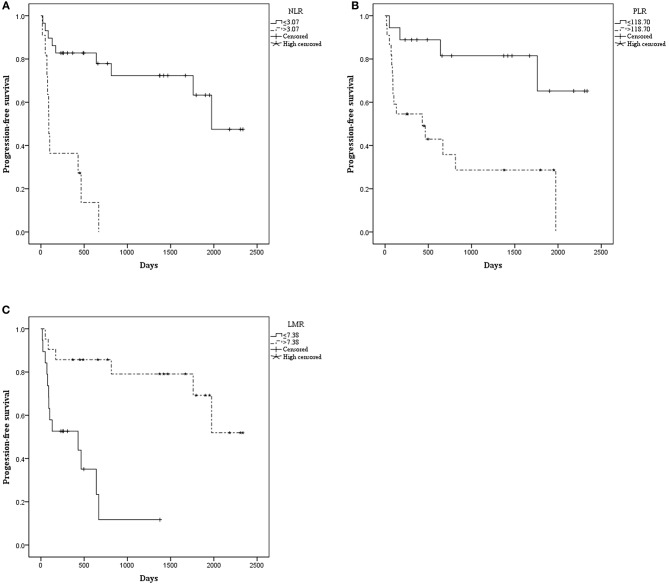
Kaplan–Meier curve for progression-free survival (PFS) of 40 patients with pretreated mucosal melanoma stratified by inflammatory makers: **(A)** PFS stratified by NLR; **(B)** PFS stratified by PLR; **(C)** PFS stratified by LMR.

The median OS was 515.00 days (95% CI: 368.00–1154.50). The median PFS was 476.50 days (95% CI: 260.00–792.00). Patients with NLR ≤ 3.07 had a significantly longer mean OS [1833.52 (95% CI: 1503.530–2163.518) vs. 366.970 (95% CI: 106.020–627.919), *P* < 0.001] (Figure 1) and mean PFS [1672.512 (95% CI: 1320.916–2024.109) vs. 238.909 (95% CI: 92.371–385.447), *P* < 0.001] (Figure 2). Likewise, PLR ≤ 118.70 was correlated with a significantly longer mean OS [2069.167 (95% CI: 1723.178–2415.155) vs. 935.562 (95% CI: 525.569–1345.556), *P* < 0.001] and mean PFS [1869.281 (95% CI: 1471.881–2266.682) vs. 756.849 (95% CI: 378.520–1135.177), *P* = 0.002]. In contrast, the group with LMR > 7.38 had a significantly longer mean OS [2102.812 (95% CI: 1838.387–2367.237) vs. 498.788 (95% CI: 270.279–727.296), *P* < 0.001] and mean PFS [1796.932 (95% CI: 1428.624–2165.241) vs. 426.754 (95% CI: 205.038–648.471), *P* < 0.001] (Table 2).

**Table 2:** Kaplan–Meier analyses of OS and PFS in 40 patients with preoperative mucosal melanoma.

		OS^*^			**PFS^‡^**		
		**Mean OS**	**95% CI^§^**	***P*-value**	**Mean PFS**	**95% CI^******§**^**	***P*-value**
**NLR^||^**	≤ 3.07	1833.524	1503.530–2163.518	<0.001	1672.512	1320.916–2024.109	<0.001
	>3.07	366.970	106.020–627.919		238.909	92.371–385.447	
**PLR^¶^**	≤ 118.70	2069.167	1723.178–2415.155	<0.001	1869.281	1471.881–2266.682	0.002
	>118.70	935.562	525.569–1345.556		756.849	378.520–1135.177	
**LMR^#^**	≤ 7.38	498.788	270.279–727.296	<0.001	426.754	205.038–648.471	<0.001
	>7.38	2102.812	1838.387–2367.237		1796.932	1428.624–2165.241	

* OS, overall survival;

‡ PFS, progression-free survival;

§ CI, confidence interval;

|| NLR, neutrophil-to-lymphocyte ratio;

¶ PLR, platelet-to-lymphocyte ratio;

# *LMR, lymphocyte-to-monocyte ratio*.

### Univariate and Multivariate Analysis

Univariate analysis showed that factors including surgery, ALP level, and NLR, LPR, and LMR levels were correlated with OS and with PFS (*P* < 0.05); thus, these factors were all included in multivariate analysis. Moreover, age (15) and metastasis (15, 16) status were reported to be associated with prognosis of other melanoma and thus underwent multivariate analysis as potential confounding factors, too. Results suggested that higher baseline LMR (>7.38) was observed to be significantly associated with OS (HR: 0.113, 95% CI: 0.017–0.772, *P* = 0.026) and performing surgery had a positive impact on OS (HR: 0.166, 95% CI: 0.033–0.846, *P* = 0.031), which meant that patients having a higher baseline LMR and surgery resulted in longer survival time with risk of death at 11.3 and 16.6%, respectively, compared to lower LMR and no surgery. Other factors were not significantly associated with OS or PFS in multivariate analysis (Table 3).

**Table 3:** Univariate and multivariate COX hazard regression test of factors associated with OS and PFS in 40 patients with preoperative mucosal melanoma.

		PFS^*^						**OS^‡^**					
		**Univariate COX hazard regression test**			**Multivariate COX hazard regression test**			**Univariate COX hazard regression test**			**Multivariate COX hazard regression test**		
	***n* (%)**	***P*-value**	**HR^§^**	**95% CI^||^**	***P*-value**	**HR^§^**	**95% CI^||^**	***P*-value**	**HR^§^**	**95% CI^||^**	***P*-value**	**HR^§^**	**95% CI^||^**
**Gender**, ***n*** **(%)**													
Male	14 (35.0)	0.212	1	/				0.101	1	/			
Female	26 (65.0)		0.546	0.212–1.411					0.437	0.162–1.176			
**Age**, ***n*** **(%)**													
<65	26 (65.0)	0.537	1	/	0.721	1	/	0.606	1	/	0.402	1	/
≥65	14 (35.0)		0.721	0.255–2.036		1.260	0.355–4.471		0.756	0.262–2.182		1.856	0.403–7.893
**Surgery**, ***n*** **(%)**													
No	4 (10.0)	0.002	1	/	0.077	1	/	0.001	1	/	0.031	1	/
Yes	36 (90.0)		0.152	0.047–0.500		0.278	0.067–1.148		0.129	0.037–0.444		0.166	0.033–0.846
**Metastasis**, ***n*** **(%)**													
No	21 (52.5)	0.182	1	/	0.481	1	/	0.216	1	/	0.109	1	/
Yes	29 (47.5)		1.888	0.742–4.805		0.649	0.195–2.157		1.880	0.692–5.109		0.264	0.052–1.346
**Chemotherapy**, ***n*** **(%)**													
No	26 (65.0)	0.243	1	/				0.159	1	/			
Yes	14 (35.0)		1.713	0.694–4.230					1.986	0.765–5.158			
**Radiotherapy**, ***n*** **(%)**													
No	31 (77.5)	0.512	1	/				0.695	1	/			
Yes	9 (22.5)		0.690	0.228–2.087					0.798	0.259–2.457			
**Hb**, ***n*** **(%)**													
< LLN	4 (10.0)	0.424	1	/				0.124	1	/			
≥LLN	36 (90.0)		0.602	0.173–2.091					0.407	0.130–1.281			
**PLT**, ***n*** **(%)**													
<300	36 (90.0)	0.080	1	/				0.074	1	/			
≥300	4 (10.0)		3.087	0.874–10.898					3.165	0.894–11.211			
**Alb**, ***n*** **(%)**													
<35	37 (92.5)	0.556	1	/				0.486	1	/			
≥35	3 (7.5)		1.559	0.356–6.839					1.701	0.382–7.572			
**ALP (UI/L)**, ***n*** **(%)**													
<150	38 (95.0)	0.009	1	/	0.202	1	/	0.006	1	/	0.131	1	/
≥150	2 (5.0)		7.935	1.676–37.570		3.146	0.540–18.318		9.190	1.896–44.538		4.567	0.637–32.714
**LDH (UI/L)**, ***n*** **(%)**													
<245	36 (90.0)	0.806	1	/				0.667	1	/			
≥245	4 (10.0)		1.203	0.276-−5.254					1.386	0.314–6.122			
**NLR^¶^**, ***n*** **(%)**													
≤ 3.07	29 (72.5)	<0.001	1	/	0.091	1	/	<0.001	1	/	0.115	1	/
>3.07	11 (27.5)		8.29	2.894–23.751		3.049	0.838–11.093		9.531	3.300–27.526		2.819	0.776–10.239
**PLR^#^**, ***n*** **(%)**													
≤ 118.70	18 (45.0)	0.005	1	/	0.353	1	/	0.004	1	/	0.199	1	/
>118.70	22 (55.0)		4.887	1.603–14.902		1.953	0.476–8.017		8.835	2.009–38.857		3.198	0.542–18.876
LMR^**^, ***n*** **(%)**													
≤ 7.38	19 (47.5)	0.001	1	/	0.189	1	/	<0.001	1	/	0.026	1	/
>7.38	21 (52.5)		0.135	0.01–0.442		0.346	0.071–1.689		0.059	0.013–0.270		0.113	0.017–0.772

* PFS, progression-free survival;

‡ OS, overall survival;

§ HR, hazard ratio;

|| CI, confidence interval;

¶ NLR, neutrophil-to-lymphocyte ratio;

# PLR, platelet-to-lymphocyte ratio;

** *LMR, lymphocyte-to-monocyte ratio*.

## Discussion

By extracting data from clinical records of the host institutions, we were able to perform analyses of mucosal melanoma, a less common type of malignant tumor accounting for 0.03% of newly diagnosed cancers (2). This is the first study comparing outcomes of different NLR, PLR, and LMR levels in preoperative mucosal melanoma. Our study demonstrated that baseline NLR > 3.07, PLR > 118.70, and LMR ≤ 7.38 are markers for aggressive tumor and were associated with poor OS. In addition, surgery is a beneficial factor associated with survival. Similarly observed with many other tumors (17–20), higher ALP was connected with poor prognosis. Meanwhile, the lack of association between NLR, PLR, and survival in multivariate analysis is believed to stem from most every patient receiving the same standardized therapies, causing diverse immune reactions among patients. Limited samples also contributed to potential bias. Additionally, relatively short follow-up time in this research, <5 years of the median time, might lead to less clinical outcomes being observed when research ended, which brings to weakened relevance between NLR, PLR, and survival. Overall, analytic results suggested that these three inflammatory markers were statistically significant prognostic indicators of survival of mucosal melanomas, especially the LMR.

The important role of inflammation in tumor initiation, progression, and metastasis is now widely accepted, and is thought to be related to the activation of neutrophils and the defect in homeostasis among immune cell components (3, 21–23). Among those inflammatory parameters, the NLR had been proposed as a marker for predicting prognosis in different tumors, such as colorectal cancer (24), urothelial carcinoma (25), renal cell carcinoma (26), lung adenocarcinoma (7), and breast cancer (27). This finding was confirmed by a systematic review (6). The PLR was also associated with prognosis of some cancers, including non-small cell lung cancer (28), urothelial carcinoma (29), biliary tract cancer (30), colorectal cancer (31), and gastric cancer (32), confirmed also by a systematic review (33). Similarly, LMR was proposed to be a prognostic predictor of various tumors, including the malignant melanoma (34).

For melanoma, the published literature indicated that both preoperative (9, 13, 35, 36) and on-therapy (11, 37, 38) periphery blood inflammatory markers were correlated with the prognosis of patients with melanoma at every stage, who are mainly receiving immunotherapy. However, our findings were only partially in accordance with previous reported literature, because no prognostic analysis has been conducted in mucosal melanoma until now as it is a unique subtype of melanoma (2). Compared with other types, mucosal melanoma is likely to have more chromosomal structural aberrations and less mutational burden (39), and it has a more aggressive performance and a worse outcome (40). Moreover, some literature has excluded mucosal melanoma (35, 41). Therefore, we believe that it is necessary to assess the relationship between inflammatory markers and the prognosis of mucosal melanoma, even though it has been reported that NLR, PLR, and LMR are potential prognostic factors of the outcome of melanoma (7, 13, 41).

We acknowledge that there are several limitations in our research, including potential bias due to the nature of retrospective research, and the relatively small number of subjects because of the rarity of this disease. Moreover, clinical and laboratory information were unavailable for some of the patients; thus, we cannot include those patients in the final analysis. Furthermore, we were not able to document more specific tumor performance status due to the lack of full-scale information, such as the tumor stage and the overall period of different therapies, which might provide more significant indication in analysis. Nevertheless, our study is still noteworthy because we are able to firstly identify the prognostic value of NLR, PLR, and LMR in mucosal melanoma, which indicate that a higher baseline NLR, PLR, and a lower baseline LMR are correlated with an unsatisfied prognosis. In addition, this study provides a basis for future research that predicts prognosis using circulation inflammatory markers and to validate a determined threshold for each marker. A blood test is the routine of clinical practice and those markers are easy to monitor without additional expenditure; therefore, we suggest prospective clinical trials be conducted to perform a more robust analysis.

## Conclusions

Preoperative peripheral inflammatory markers (NLR, PLR, and LMR) were indicators of prognosis in patients with mucosal melanoma. NLR > 3.07, PLR > 118.70, and LMR ≤ 7.38 were validated in our study to be correlated with poorer OS and PFS.

## Data Availability Statement

The datasets generated for this study are available on request to the corresponding author.

## Author Contributions

Conceptualization, writing—review and editing, and Supervision: YW and HZ. Methodology and investigation: YY. Software, formal analysis, and writing—original draft preparation: YW. Validation: YW, YY, TZ, HZ, and XM. Resources and project administration: XM. Data curation: TZ. All authors contributed to manuscript revision, and read and approved the submitted version.

### Conflict of Interest

The authors declare that the research was conducted in the absence of any commercial or financial relationships that could be construed as a potential conflict of interest.

## Abbreviations

CRP: C-reactive protein
NLR: neutrophil-to-lymphocyte ratio
LMR: lymphocyte-to-monocyte radio
LDH: lactate dehydrogenase
PLR: platelet-to-lymphocyte ratio
Hb: hemoglobin
Alb: albumin
ALP: alkaline phosphatase
LDH: lactate dehydrogenase
OS: overall survival
PFS: progression-free survival
ROC: receiver operating characteristic.
